# Synthesis and Integration of Hybrid Metal Nanoparticles Covered with a Molecularly Imprinted Polymer Nanolayer by Photopolymerization

**DOI:** 10.3390/s23083995

**Published:** 2023-04-14

**Authors:** Amine Khitous, Céline Molinaro, Constance Thomas, Karsten Haupt, Olivier Soppera

**Affiliations:** 1 Université de Haute-Alsace, CNRS, IS2M UMR 7361, F-68100 Mulhouse, France; amine.khitous@uha.fr (A.K.);; 2 Université de Strasbourg, F-67081 Strasbourg, France; 3 Université de Technologie de Compiègne, CNRS Laboratory for Enzyme and Cell Engineering, F-60203 Compiègne, France

**Keywords:** molecularly imprinted polymer, plasmonic, SERS, chemical sensor

## Abstract

Interfacing recognition materials with transducers has consistently presented a challenge in the development of sensitive and specific chemical sensors. In this context, a method based on near-field photopolymerization is proposed to functionalize gold nanoparticles, which are prepared by a very simple process. This method allows in situ preparation of a molecularly imprinted polymer for sensing by surface-enhanced Raman scattering (SERS). In a few seconds, a functional nanoscale layer is deposited by photopolymerization on the nanoparticles. In this study, the dye Rhodamine 6G was chosen as a model target molecule to demonstrate the principle of the method. The detection limit is 500 pM. Due to the nanometric thickness, the response is fast, and the substrates are robust, allowing regeneration and reuse with the same performance level. Finally, this method of manufacturing has been shown to be compatible with integration processes, allowing the future development of sensors integrated in microfluidic circuits and on optical fibers.

## 1. Introduction

Due to their localized surface plasmon (LSPR) properties, metallic nanoparticles (MNPs) are nanoobjects of great interest in many fields such as photonics, molecular detection [1,2,3] or photocatalysis for various reactions [4,5]. Due to the nanometric size of nanoparticles (NPs), resonance phenomena cause an exaltation of the electromagnetic field in the vicinity of metallic nanostructures (optical near field, ONF), which considerably increases the coupling with molecules present on their surface and thus amplifies their optical response [6]. This principle allows the significant increase of the Raman signal by offering surface-exalted Raman spectrometry (SERS) [7,8]. In addition to SERS, near-field fluorescence may also be increased under certain conditions [9]. Another interesting property is the sensitivity of the optical properties of MNPs towards changes in the refractive index of their environment. This allows probing target molecules that accumulate in the vicinity, for example, upon their recognition by specific sites or cavities in the sensing layer [4,6,10], thus providing opportunities for applications in the sensor field.

For these applications, a key step is the functionalization of the MNP surface with a recognition layer. Various MNP functionalization methods have been proposed; one of them is the functionalization by self-assembled monolayers (SAMS) [11], but this method requires a multistep process and is not localized. The use of aptamers or DNA [12,13] may provide very precise functionalization, but is limited to very specific conditions that avoid, for example, temperatures higher than 38 °C or saline environments, both of which can cause denaturation and loss of the 3D biomolecular structure and, thus, recognition [14].

Among the materials capable of withstanding harsh analytical conditions (temperature, pressure, and salinity of the medium) are molecularly imprinted polymers (MIPs). MIPs are materials that have achieved great success in the analytical community due to their ease of fabrication and robustness [14]. They consist of a polymer network containing recognition sites for a target molecule. To prepare them, a target molecule is selected and used as a molecular template, which is allowed to form a complex with suitable monomers that have a good affinity for the template through reversible interactions (van der Waals forces, ionic or hydrogen bonds, for example), thus offering a wide choice of complexing agents. Crosslinker monomers are added and copolymerized using a suitable initiator, thus ‘freezing’ the 3D complex in space. Finally, the resulting polymer is processed in order to weaken the interactions between the template and the polymer to liberate the binding cavities that have formed [6,15,16,17]. The MIP is now able to specifically bind its target molecule.

The method of polymer synthesis plays a key role in the recognition properties of the target molecule by the MIP. For example, thermopolymerization of MIPs can lead to a weakening of the molecular interactions between the target and the complexing agent [14], which can reduce and affect the recognition sites. For this reason, the photochemical route (by photopolymerization) is attractive in the preparation of MIPs, as lower temperatures can be used. The thickness of the MIP layer is another crucial factor. The thicker the MIP, the greater the probability of buried sites in the MIP that are unable to release the template, which can interfere with the analysis [6,18,19] and are unavailable for sensing. Even in the case of porous MIPs, thick films can slow down mass transfer and show significant swelling, which can result in the loss of recognition sites, thereby reducing detection sensitivity [18]. Again, the control of film thickness is easier with photo rather than thermopolymerization [20].

To circumvent these limitations and simplify the integration of hybrid nanoparticles, a breakthrough synthesis method has been proposed, namely near-field photopolymerization (NFP). This method is based on the interaction of light with AuNPs that induces near-field light exaltation via LSPR. This allows local photopolymerization around the MNPs where light is exalted [6,21,22]. To avoid far-field polymerization, the threshold dose for far-field polymerization (without MNPs) is determined; once determined, a drop of this formulation was deposited on the surface of MNPs and irradiated at doses below the threshold dose to avoid bulk polymerization [21,22]. Due to the light enhancement in the near field, the light intensity near the MNPs is higher than in the far field, allowing us to initiate polymerization only on the surface of the MNPs (at doses below the threshold dose). This method allows us to locally integrate the MNPs with a functional MIP layer and to control the polymerized thickness at the nanoscale.

In this work, AuNPs synthesized by thermal dewetting were used, a method that has raised great interest in preparing surfaces coated with non-aggregated and high density MNPs [4,6,23]. These substrates have been used in the spectral monitoring of near-field polymerization. Recently, the same method was applied to synthesize a specific MIP for methylene blue (MB) with good sensitivity and selectivity [6]. In order to show the interest and universality of this synthesis method, in this work, Rhodamine 6G (R6G) was chosen as the target by changing the formulation and the type of photoinitiator. For the preparation of MIPs, methacrylic acid (MAA) was used as the complexing agent (functional monomer), pentaerythritol triacrylate (PETA) and ethylene glycol dimethyl acrylate (EGDMA) as cross-linking monomers, and the eosin Y (EY)–N-methyldiethanolamine (MDEA) couple as photoinitiator (Norrish II system) [22]. There must be a spectral overlap between the AuNPs and the photoinitiator in order to achieve ONF photopolymerization. For example, in this study, AuNPs and EY (photoinitiator) absorb light at 532 nm. AuNPs can enhance the efficiency of the photoinitiator and accelerate ONF photopolymerization. The latter was monitored by UV-vis and SERS spectrometry and verified by TEM. To ensure that the recognition was due to the imprinted sites and not due to nonspecific interactions between the polymer and R6G, a control polymer (NIP) was prepared by removing the target (R6G) from the MIP formulation during photopolymerization. SERS was chosen as a detection method because it is an efficient technique, relatively simple to implement and sensitive; and it verifies the structure of the detected molecule through its spectral signature [24]. The study of detection sensitivity and recognition was performed by SERS. For the selectivity study, MB and rhodamine 110 (R110) were chosen as analogues due to their similar structure to R6G [6]. Two examples of the integration of these nanoparticles on an optical fiber and in a microfluidic device illustrate the interest of this method and provide opportunity for a much simpler use of these functionalized nanoparticles.

## 2. Material and Methods

### 2.1. Synthesis of AuNPs by Thermal Dewetting

**Figure 1:**
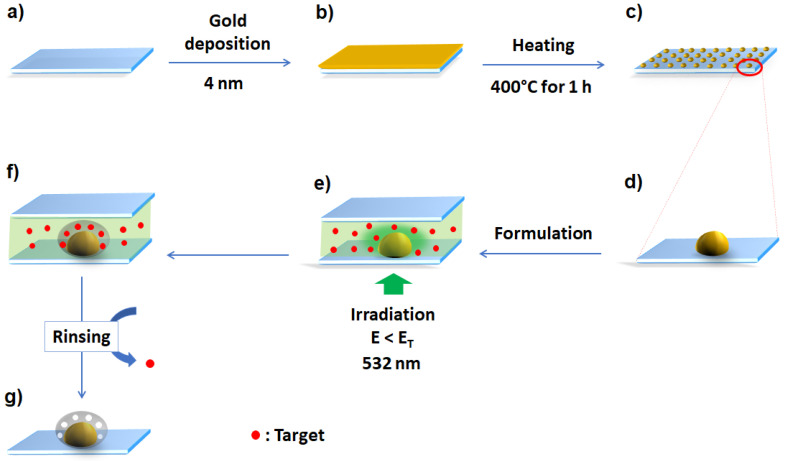
Protocol for the synthesis of AuNPs@MIP-R6G by NFP: (**a**): preparation of the glass slide, (**b**): deposition of a 4 nm gold film, (**c**): thermal dewetting at ’400 °C for 1 h, (**d**): zoom on a nanoparticle, (**e**) deposition of the photopolymerizable MIP formulation and irradiation of the whole system at 532 nm, (**f**) synthesis of the AuNPs@MIP-R6G by localized photopolymerization, and (**g**) recognition sites are revealed after washing.

The protocol for the synthesis of hybrid AuNPs is shown in Figure 1. AuNPs were synthesized by thermal dewetting on glass slides (22 × 22 mm^2^ CARL ROTH, Karlsruhe, Germany), which were first rinsed in acetone (10 min) under ultrasound (Bandelin SONOREX Ultrasonic bath, Berlin, Germany), then in ethanol (10 min) under ultrasound and dried under a flow of air. These slides were then heated in an oven at 100 °C for 15 min and treated with UV-Ozone (UV/ozone, Procleaner Plus, Bioforce Nanoscience, IA, USA) for 20 min. After cleaning, a 4 nm gold layer was deposited by cathodic evaporation (sputtered by a CRESSINGTON 108 auto, Microscopy & Imaging Center, TX, USA). Finally, the gold films deposited on the glass slide were heated at 400 °C for 1 h in ambient air (GESTIGKEIT-PR 5 3T hot plate, The laboratory store, Inverness-shire, UK) to obtain AuNPs absorbing around 532 nm.

### 2.2. Synthesis of AuNPs@MIP-R6G

A formulation of MIP-R6G was prepared as follows: First, a mixture of rhodamine 6 G (R6G, C_28_H_31_N_2_O_3_Cl, 99% purity, Sigma Aldrich, Munich, Germany) and ethylene glycol dimethacrylate (EGDMA, C_10_H_14_O_4_, 98% purity, Sigma Aldrich, Munich, Germany) was prepared and stirred for 20 min, sonicated for 10 min and then stirred for 2 h. Finally, pentaerythritol triacrylate (PETA, C_14_H_18_O_7_, 298.29 g.mol^−1^, 97% purity, Sigma Aldrich, Munich, Germany), N-methyl diethanolamine (MDEA, CH_3_N(CH_2_CH_2_OH)_2_, 99% purity, Sigma Aldrich, Munich, Germany) and eosin Y (EY, C_20_H_6_Br_4_Na_2_O_5_, 99% purity, Sigma Aldrich, Munich, Germany) were added. The ratios of this formulation in mass wt% were (R6G:MAA:PETA:EGDMA:EY:MDEA = 0.34:22.45:52.57:18.60:0.38:5.66). The formulation was stirred in the dark for 2 h and was purged with nitrogen for 10 min before use.

The threshold dose (D_t_) is the minimum energy required to induce far-field photopolymerization. To determine D_t_, a drop of the formulation was placed between two glass slides (22 × 22 mm^2^ CARL ROTH, Karlsruhe, Germany) and irradiated at 532 nm at different times with a Verdi 6 W Coherent laser at a power of 25 mW/cm^2^; the latter was measured with a powermeter (Thorlabs PM100 Power Meter S120C, Maisons-Laffitte, France). After irradiation, the slides were delaminated to check for far-field polymerization with the naked eye. The threshold time for this polymerization was 30 s, corresponding to a D_t_ of 750 mJ.

Once the threshold dose was determined, a drop of this formulation was deposited on the AuNPs and covered with a glass slide. The assembly was irradiated at 90% E_T_ (corresponding to 27 s at 25 mW/cm^2^). This allows for local photopolymerization only where the light is exalted.

### 2.3. Synthesis of AuNPs@NIP

To prepare a non-imprinted polymer (NIP), R6G was omitted from the formulation to obtain a chemically identical polymer without recognition sites. The preparation protocol and the determination of the threshold dose of NIP were performed according to the same protocol as for MIP. Once the threshold dose was determined, a drop of this solution was irradiated at 532 nm under the same conditions as for MIP (above).

### 2.4. Study of the Recognition Efficiency of AuNPs@MIP-R6G

After synthesis and to reveal the recognition sites, the AuNPs@MIP-R6G were rinsed in pure ethanol, under stirring at 300 rpm for 10 min, then 3 times in methanol: acetic acid (90:10) for 10 min and once in pure methanol. Target recognition was first investigated by UV-vis spectrometry by incubating MIP and NIP in a concentration range of 5–100 nM of R6G in ethanol. The MIP and NIP were then studied by SERS in the same solutions. After incubation, the samples were rinsed 3 times in pure ethanol to remove adsorbed molecules. In particular, we showed in a control experiment that bare AuNPs incubated in 100 nM R6G and rinsed 3 times in pure ethanol showed no SERS signal from R6G. This shows that adsorbed R6G can be removed from AuNPs with such protocol.

### 2.5. Characterization Instruments

The AuNPs@MIP-R6G were characterized using a fiber optic UV-Vis spectrophotometer (AvaSpec-ULS2048CL-EVO, Avantes, Apeldoorn, The Netherlands), a Raman spectrometer under 532 nm excitation (Labram 300 spectrophotometer, Horiba, Palaiseau, France) and a high-resolution transmission electron microscope (NEOARM/JEM-ARM200F, JEOL, Croissy-sur-Seine, France). For HRTEM analysis, the same protocol as for the synthesis of AuNPs@MIP-R6G on a glass slide was used to synthesize AuNPs@MIP-R6G on a silicon TEM grid with two rectangular windows (1500 µm × 100 µm) covered by a 50 nm thick Si_3_N_4_ membrane (Ted Pella, Roissy, France).

## 3. Results and Discussion

**Figure 2:**
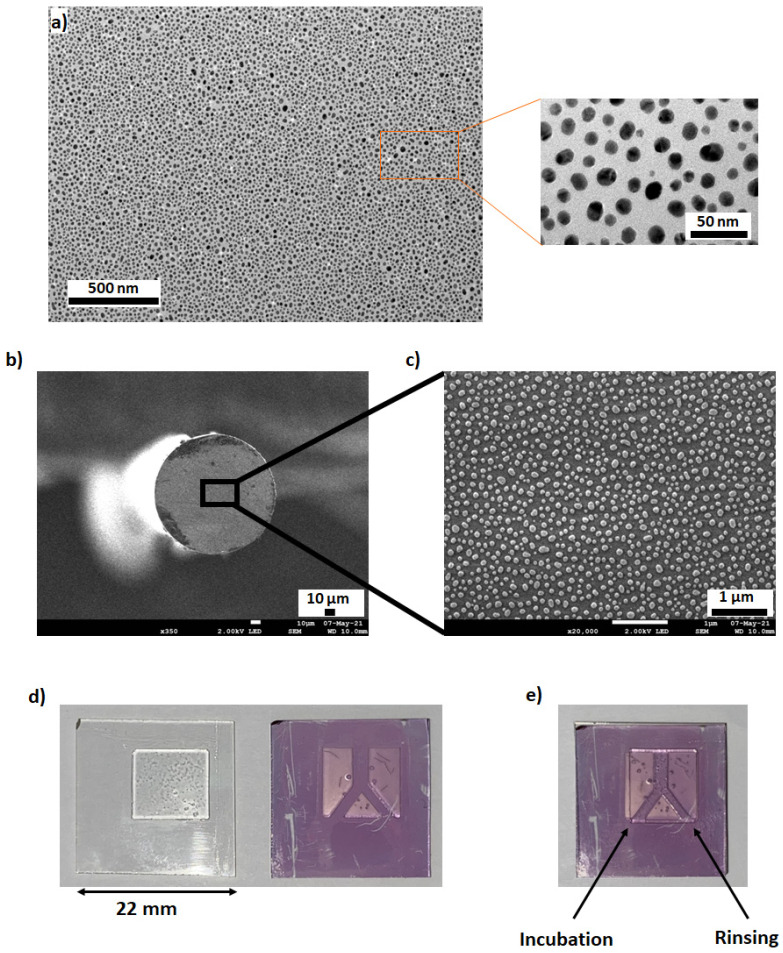
(**a**): TEM image of AuNPs synthesized by thermal dewetting from a 4 nm gold film on a glass substrate at 400 °C for 1 h, (**b**): synthesis of AuNPs by thermal wetting at the end of a 780 HP optical fiber of 125 µm diameter, (**c**): zoom on the optical fiber, (**d**): integration of a fluidic circuit on an AuNPs substrate by 3D printing (**right**) and its cover (**left**), (**e**): sealing of the fluidic circuit by live photopolymerization, the arrows indicate the rinsing and incubation circuits.

Based on previous studies [4,6,24], the AuNPs used in this work were synthesized by thermal dewetting of a 4 nm gold film deposited on a glass slide (Figure 1). The increase in temperature results in the melting of the gold deposit [4,25]. For these low thicknesses, the molten gold film can de-wettedthrough regimes of nucleation and spinodal decomposition, depending on its thickness, to give rise to nanoparticles with a nearly homogenous size distribution under certain conditions [25]. In this work, after heating at 400 °C for 1 h, the film turns into well-dispersed AuNPs (Figure 2), without aggregates and with high density over the entire substrate surface, which is one of the advantages of this synthesis method. In previous work and using the same AuNPs, analysis of SEM, TEM and AFM images showed an oval shape of the AuNPs with an average diameter of 15 ± 7 nm and a height of 10 ± 4 nm [4,26].

The interest of this method of integrating AuNPs is that it can also be easily applied to different types of substrates. Figure 2 shows how this approach can be used to obtain AuNPs at the end of an optical fiber tip or in a microfluidic circuit prepared by UV 3D printing. The nanometer-thick film was deposited in the same way as on the glass substrate. Then, a thermal treatment at 600 °C for 2 h generated the AuNPs visible on the SEM image. Note that this thermal annealing step can be replaced by a fast and easily applicable NIR laser treatment on plastic fibers [24]. It is then very simple to apply the principle of near-field photopolymerization by injecting light into the fiber and generating the MIP layer around the AuNPs.

Figure 2d,e also shows the 3D printing of a basic fluidic circuit on the substrate containing the AuNPs. In this case, the AuNPs@MIPs were first generated on a glass substrate, and then a UV resin was deposited and UV-cured to generate the microfluidic circuit. The rest of this study concerns AuNPS@MIP synthesized on this type of substrate.

**Figure 3:**
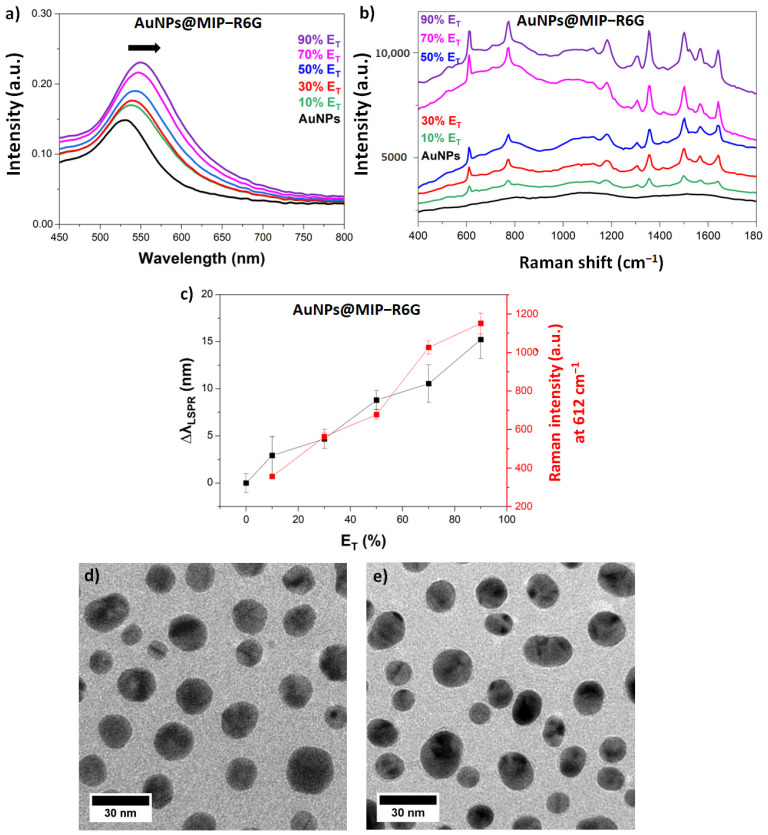
Monitoring of photopolymerization in ONF by (**a**) UV-vis spectrometry and (**b**) SERS; (**c**): Plot of LSPR band shift and SERS signal intensity increase from R6G to 612 cm^−1^ as a function of threshold energy (E_T_%), TEM images of AuNPs before (**d**) and after (**e**) irradiation at 90% E_T_.

As can be seen in Figure 3a, AuNPs have a resonance at 532 nm, which justifies the use of a 532 nm absorbing photopolymer and, in particular, the choice of Eosin Y as a sensitizer. The photopolymerization in ONF was followed as a function of the irradiation dose by UV-vis and SERS spectroscopy.

Figure 3a shows the monitoring of photopolymerization by UV-vis spectrometry by the LSPR band shift of AuNPs. The spectra are recorded after rinsing the AuNPs after each irradiation to remove the non-polymerized monomers. The spectrum of the rinsed AuNPs after contact with the formulation and without irradiation shows no shift in the LSPR band (Figure S1). After irradiation, a shift in the LSPR band is observed which is due to the increased effective refractive index around the AuNPs during ONF. The increase of the irradiation dose (between 10 and 90% E_T_) induces an increase in the LSPR shift explained by the increase of the MIP thickness around the AuNPs (Figure S2). Following the photopolymerization by SERS showed the same trend. Increasing the irradiation dose induces an increase in the R6G signal present in the MIP (Figure 3b). The spectral shift of the LSPR and the intensity of the band at 612 cm^−1^ in the Raman spectrum follow the same trend (Figure 3c). TEM was used to confirm the presence of MIP on the surface of AuNPs. After 90% E_T_ irradiation, the TEM images show a 2 nm thin MIP layer appearing around the AuNPs (Figure 3d,e).

**Figure 4:**
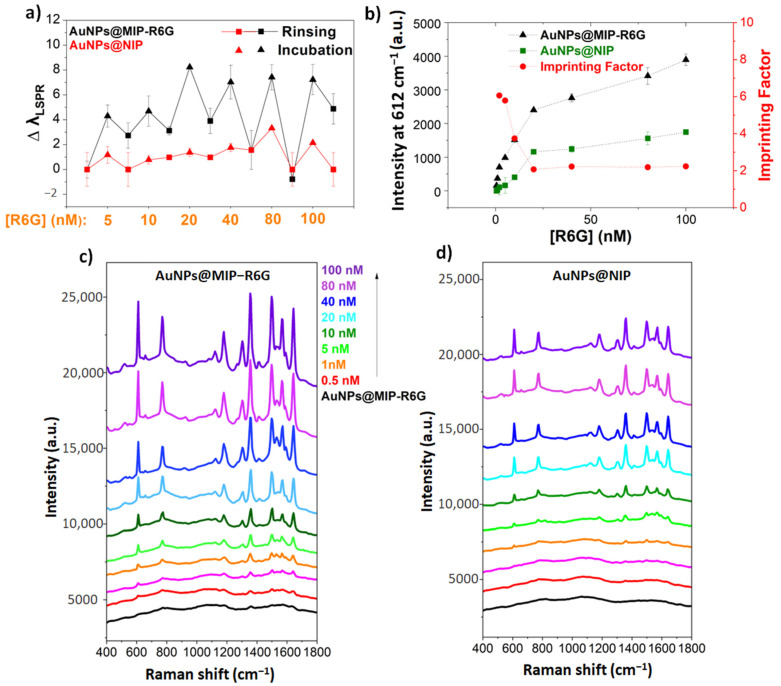
(**a**) Monitoring of R6G recognition by LSPR shift followed by UV-vis spectrophotometry in the case of MIP (black) and NIP (red), the triangles show the results after incubation and the squares after the rinses. (**b**) Plot of the SERS intensity for AuNPs@MIP-R6G and AuNPs@NIP and imprinting factor (IF) as a function of the R6G concentration (**c**,**d**) Monitoring of R6G recognition by SERS in the case of AuNPs@MIP-R6G and AuNPs@NIP respectively.

After the synthesis of AuNPs@MIP-R6G, the functionalized NPs were rinsed according to the protocol described in the experimental section, which removes the R6G molecules embedded in the polymer. Figure S2 shows that the polymer layer is firmly anchored at the surface of the particle and not affected by the washing procedure. The detection of R6G was then studied by UV-vis spectroscopy by recording the absorption spectra of the samples after incubation in solutions of different concentrations of R6G in ethanol (5 and 100 nM). The shift of the LSPR band, which is sensitive to the change in refractive index around the nanoparticles, is used to track the rebinding. Figure 4a shows the summary of detection tracking after each incubation and rinse for AuNPs@MIP-R6G and the control AuNPs@NIP. The UV-vis spectra are shown in Figure S3. To verify that the binding of R6G is due to specific recognition by imprinted sites and not just nonspecific adsorption onto the polymer surface, the AuNPs@NIP were incubated with R6G at the same concentrations. No significant LSPR shift is detected in the case of AuNPs@NIP, showing recognition related to specific imprints of the target molecule (Figure 4a).

The binding study was also conducted by SERS, for incubations in solutions of R6G in the range [0.5–100 nM] in ethanol. The results are shown in Figure 4c. It should be noted that the rinsed AuNPs@MIP-R6G do not show any R6G signal, confirming the release of recognition sites upon rinsing after incubation. This point shows the interest of the method: it integrates the functional matrix selectively on AuNPs, while controlling the thickness of the polymer layer at the nanoscale. This is a very simple method to implement that limits the buried sites, which can generate a loss of usable recognition sites or interfere with the analysis. Indeed, after rinsing the MIPs, a negligible signal remains from the target (Figure S4), demonstrating that most recognition sites are accessible by the rinsing method used.

Increasing the concentration of R6G in the solution induces an increase in the SERS signal. The limit of detection was estimated to be 500 pM. This value was confirmed by incubating the AuNPs@MIP-R6G 10 times in a 500 pM R6G solution with similar results. The corresponding spectra are shown in Figure S5.

To confirm the specific recognition with the SERS measurements, AuNPs@NIP were incubated in the same concentration range of R6G as used with AuNPs@MIP-R6G [0.5–100 nM]. Figure 4d shows that for low concentrations, negligible recognition was observed. As the concentration of R6G increases, there is an increase in non-specific adsorption. Figure 4b summarizes the peak intensities at 612 cm^−1^ of R6G recognized by AuNPs@MIP-R6G and AuNPs@NIP. The variation of the imprinting factor (IF = Intensity at 612 cm^−1^ of R6G MIP/Intensity of R6G NIP) has been plotted on the same figure. The value of IF must be higher than 3 for a good sensor [25]. It can be seen that at lower concentrations (below 20 nM), the IF is greater than 3. By increasing the concentration of R6G (above 20 nM), the IF decreases. This decrease is due to the participation of nonspecific recognition by the MIP after saturation of the specific sites. Comparing the detection sensitivity of this work with recent work [6], AuNPs@MIP-R6G has a higher sensitivity (500 pM) than AuNPs@MIP-MB (10 nM). This result can be explained by the better spectral overlap between the absorption spectrum of R6G and the extinction spectrum of AuNPs. Both are centered at 532 nm, which optimizes the SERS signal of R6G and results in a lower detection limit. In the case of methylene blue, which absorbs at 655 nm, the overlap is not as good, thus reducing its excitation and, ultimately, sensitivity in SERS compared to R6G.

**Figure 5:**
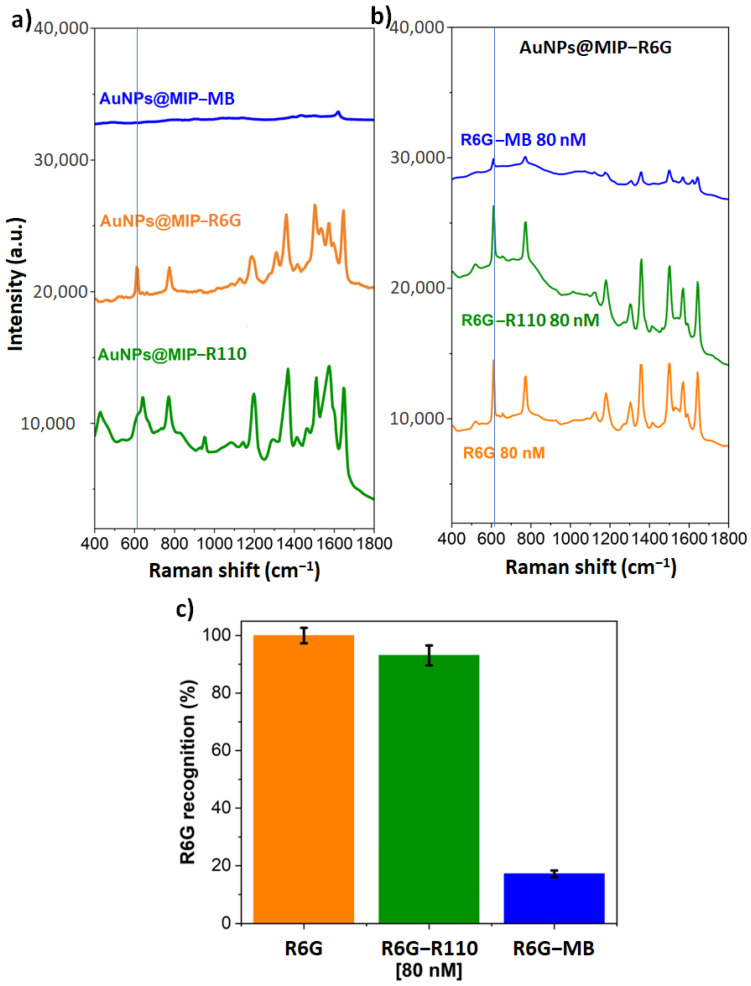
(**a**) Study of the relativity of R110, R6G and MB signals in SERS by polymerizing the same concentrations of each molecule in the near field, (**b**) study of the recognition of R6G in competition with R110 and MB at 80 Nm, (**c**) summary of the competitive recognition experiments.

Detection selectivity was also demonstrated by competitive binding tests with other analytes. The AuNPs@MIP-R6G were incubated in a solution containing two molecules with a structure close to that of R6G, namely methylene blue (MB) and Rhodamine 110 (R110). As the SERS signal depends on the effective absorption cross section of the molecule and the AuNPs, two formulations containing MB and R110, respectively, at the same concentration as R6G, were previously polymerized in ONF to compare the signals of the three molecules. This allows the same amount of molecule to be immobilised in the polymer shell to normalize and relativize the signals of the molecules used (Figure 5a). For this, the signal of R6G at 612 cm^−1^ is divided by that of MB at 1618 cm^−1^. The signal of MB represents 16% of the signal of R6G, while the signal of R110 is similar to that of R6G. The SERS spectra are shown in Figure 5a, under Raman excitation at 532 nm. It can be seen that MB, which absorbs at 655 nm, shows a weaker SERS signal than R6G and R110, which absorb at 532 nm. For this reason, the signals of the two analogues have been normalized (at 635 cm^−1^ for R110 and at 1618 cm^−1^ for MB) by dividing their signal by that of R6G at 612 cm^−1^.

Incubation in solutions containing R6G and R110 at 80 nM (Figure 5b) gives 93% expected signal for R6G. To obtain this value, the specific peak at R110, at 635 cm^−1^, is used to differentiate against R6G. Thus, there is evidence that the imprints are specific for R6G versus R110. By comparing the structure of these two molecules, it should be noted that R6G has two secondary amine groups while R110 has two primary amine groups, which may explain the difference in affinity of MIP-R6G for the two molecules (Figure S6).

Competitive binding tests between R6G and MB were also conducted. Knowing that the MB signal at 1618 cm^−1^ represents 16% of the R6G signal at 612 cm^−1^, it is observed from the spectra shown in Figure 5b that incubation of R6G with MB reduced the recognition of R6G to 17%. This shows a higher affinity of MB for MIP-R6G than for R6G. It can be noted that MB is a smaller molecule than the two others. Also, it has two tertiary amine groups, showing an amine group polarity closer to R6G than R110. In previous work [6], a MIP for MB was synthesized from a formulation using the same method; the recognition of MB was more selective. The competition of MB with R6G gave 74% of the signal of MB; this competition can be explained by the differences in the amine functions between the 3 molecules. As shown in Figure S6, MB has a tertiary amine group with CH_3_; R6G has secondary amine groups, while R110 has a more acidic primary amine group (Figure S6). Knowing that MAA complexes R6G via hydrogen bonds on the amine groups during the preparation, differences in rebinding are expected due to differences in amine function. Moreover, MB has a smaller size than R6G, which may explain its competition with R6G.

**Figure 6:**
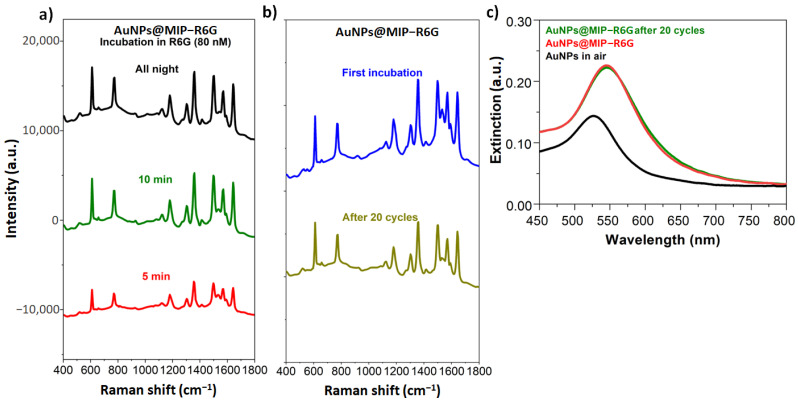
(**a**) SERS study of R6G recognition kinetics by AuNPs@MIP-R6G (80 nM), (**b**) robustness study of AuNPs@MIP-R6G by SERS via R6G recognition after 20 recognition cycles (80 nM), (**c**) robustness study of AuNPs@MIP-R6G by spectrometry via the LSPR band shift after 20 recognition cycles (80 nM).

The nanometric layer of MIP optimizes the interaction with the metallic nanostructures as shown by the excellent detection limit by SERS and also provides a fast response. The low thickness of MIP-R6G enables the majority of the recognition sites to be exposed to the surrounding environment, which results in a short recognition time. After only 5 min of incubation, the SERS signal is clearly observable. After only 10 min of incubation, the response is identical to that obtained after an overnight incubation (Figure 6a), thus demonstrating a fast response. The performance of our SERS plateform is thus competitive with those described in other recent works [27,28,29].

The other interest of these nanoparticles is their robustness: the AuNPs@MIP-R6G demonstrated stability after 20 rinse/incubation cycles, as shown in Figure 6b. The reproducibility of the SERS response shows the stability of the recognition sites. Furthermore, the LSPR band remains at the same position after these 20 rinse/incubation cycles, confirming the stability of the polymer layer (Figure 6c). This property is a fundamental characteristic to consider practical applications.

## 4. Conclusions

This study demonstrates the simplicity and efficiency of the synthesis of metal/polymer hybrid nanoparticles with molecular imprints by ONF photopolymerization. In spite of its extreme simplicity, this method allows a very fine control of the localization of the functional layer and its thickness at the nanoscale. These conditions optimize the coupling between the recognition sites and the electromagnetic hotspots of the MNPs and limit the number of recognition sites buried in the polymer that would be inaccessible to the target molecules or the rinsing solvents. Detection can be done by simple measurement of LSPR displacement after incubation, but better sensitivity is obtained by SERS. In this case, the detection limit is estimated at 500 pM, with a very good selectivity. Other key advantages of this method are a fast recognition due to the very thin MIP layers and an excellent robustness of the nanostructures due to the properties of the cross-linked acrylate-based polymer. Based on recent work [6] and this work, it has been shown that this technique offers a wide choice of target molecules, as it can be applied to other configurations, such as optical fibers, and can be integrated on a substrate with fluidic circuits to detect several molecular targets on the same substrate.

## Data Availability

Data are available upon request from the authors.

## Supplementary Materials

The following supporting information can be downloaded at: https://www.mdpi.com/article/10.3390/s23083995/s1. Figure S1: UV-vis spectra of bare AuNPs (black) and rinsed AuNPs after contact with the MIP-R6G formulation without irradiation (red). Figure S2: UV-vis spectra of bare AuNPs (black), functionalized by a layer of 90% ET MIP-R6G (green) and rinsed after functionalization (red). Figure S3: UV-vis spectra of AuNPs@MIP-R6G after incubation in R6G at different concentrations. Figure S4: SERS spectra of AuNPs, AuNPs@MIP-R6G after synthesis and washing. Figure S5: SERS spectra of AuNPs@MIP-R6G after 10 incubations in R6G at 500 pM. Figure S6: Molecular structures of: methylene blue (MB), rhodamine 6G (R6G) and rhodamine 110 (R110).

## References

[1] Szunerits S., Boukherroub R. (2012). Sensing using localised surface plasmon resonance sensors. Chem. Commun..

[2] Willets K.A., Van Duyne R.P. (2007). Localized surface plasmon resonance spectroscopy and sensing. Annu. Rev. Phys. Chem..

[3] Chen J.-S., Chen P.-F., Lin H.T.-H., Huang N.-T. (2020). A Localized Surface Plasmon Resonance (LSPR) sensor integrated automated microfluidic system for multiplex inflammatory biomarker detection. Analyst.

[4] Khitous A., Lin C.-F., Kameche F., Zan H.-W., Malval J.-P., Berling D., Soppera O. (2021). Plasmonic Au nanoparticle arrays for monitoring photopolymerization at the nanoscale. ACS Appl. Nano Mater..

[5] Tesema T.E., Kafle B., Habteyes T.G. (2019). Plasmon-driven reaction mechanisms: Hot electron transfer versus plasmon-pumped adsorbate excitation. J. Phys. Chem. C.

[6] Khitous A., Molinaro C., Gree S., Haupt K., Soppera O. (2023). Plasmon-Induced photopolymerization of molecularly imprinted polymers for nanosensor applications. Adv. Mater. Interfaces.

[7] Zhang D., You H., Zhang L., Fang J. (2020). Facile surface modification of mesoporous au nanoparticles for highly sensitive SERS detection. Anal. Chem..

[8] Du Z., Qi Y., He J., Zhong D., Zhou M. (2021). Recent advances in applications of nanoparticles in SERS in vivo imaging. Wiley Interdiscip. Rev. Nanomed. Nanobiotechnol..

[9] Yaraki M.T., Tan Y.N. (2020). Metal nanoparticles-enhanced biosensors: Synthesis, design and applications in fluorescence enhancement and surface-enhanced raman scattering. Chem. Asian J..

[10] Hong Y.A., Ha J.W. (2022). Enhanced refractive index sensitivity of localized surface plasmon resonance inflection points in single hollow gold nanospheres with inner cavity. Sci. Rep..

[11] Lee W.-I., Subramanian A., Mueller S., Levon K., Nam C.-Y., Rafailovich M.H. (2022). Potentiometric biosensors based on molecular-imprinted self-assembled monolayer films for rapid detection of influenza a virus and SARS-CoV-2 spike protein. ACS Appl. Nano Mater..

[12] Mahmoud A.M., Alkahtani S.A., Alyami B.A., El-Wekil M.M. (2020). Dual-recognition molecularly imprinted aptasensor based on gold nanoparticles decorated carboxylated carbon nanotubes for highly selective and sensitive determination of histamine in different matrices. Anal. Chim. Acta.

[13] Xu G., Hou J., Zhao Y., Bao J., Yang M., Fa H., Yang Y., Li L., Huo D., Hou C. (2019). Dual-signal aptamer sensor based on polydopamine-gold nanoparticles and exonuclease I for ultrasensitive malathion detection. Sens. Actuators B Chem..

[14] Uzun L., Turner A.P. (2016). Molecularly-imprinted polymer sensors: Realising their potential. Biosens. Bioelectron..

[15] Fuchs Y., Soppera O., Haupt K. (2012). Photopolymerization and Photostructuring of molecularly imprinted polymers for sensor applications—A review. Anal. Chim. Acta.

[16] Haupt K., Mosbach K. (2000). Molecularly imprinted polymers and their use in biomimetic sensors. Chem. Rev..

[17] Mier A., Maffucci I., Merlier F., Prost E., Montagna V., Ruiz-Esparza G.U., Bonventre J.V., Dhal P.K., Tse Sum Bui B., Sakhaii P. (2021). Molecularly imprinted polymer nanogels for protein recognition: Direct proof of specific binding sites by solution STD and WaterLOGSY NMR spectroscopies. Angew. Chem. Int. Ed..

[18] Sellergren B., Allender C.J. (2005). Molecularly imprinted polymers: A bridge to advanced drug delivery. Adv. Drug Deliv. Rev..

[19] Sellergren B. (1994). Direct drug determination by selective sample enrichment on an imprinted polymer. Anal. Chem..

[20] Kameche F., Heni W., Telitel S., Ge D., Vidal L., Dumur F., Gigmes D., Lalevee J., Marguet S., Douillard L. (2020). Plasmon-triggered living photopolymerization for elaboration of hybrid polymer/metal nanoparticles. Mater. Today.

[21] Kameche F., Heni W., Telitel S., Vidal L., Marguet S., Douillard L., Fiorini-Debuisschert C., Bachelot R., Soppera O. (2021). Probing plasmon-induced chemical mechanisms by free-radical nanophotopolymerization. J. Phys. Chem. C.

[22] Deeb C., Ecoffet C., Bachelot R., Plain J., Bouhelier A., Soppera O. (2011). Plasmon-based free-radical photopolymerization: Effect of diffusion on nanolithography processes. J. Am. Chem. Soc..

[23] Lin C.-F., Khitous A., Zan H.-W., Soppera O. (2021). Exploiting thermoplasmonic effects for laser-assisted preparation of Au nanoparticles/InZnO thin film with visible range photodetection properties. Adv. Opt. Mater..

[24] Szymańska-Chargot M., Cybulska J., Zdunek A. (2011). Sensing the structural differences in cellulose from apple and bacterial cell wall materials by raman and FT-IR spectroscopy. Sensors.

[25] Fuchs Y., Linares A.V., Mayes A.G., Haupt K., Soppera O. (2011). Ultrathin Selective Molecularly Imprinted Polymer Microdots Obtained by Evanescent Wave Photopolymerization. Chem. Mater..

[26] Mao S., Liu J., Pan Y., Lee J., Yao Z., Pandey P., Kunwar S., Zhu Z., Shen W., Belfiore L.A. (2019). Morphological and optical evolution of metallic oxide/Au nanoparticle hybrid thin film: High absorption and reflectance by plasmonic enhancement. Appl. Surf. Sci..

[27] Arabi M., Ostovan A., Zhang Z., Wang Y., Mei R., Fu L., Wang X., Ma J., Chen L. (2021). Label-free SERS detection of raman-inactive protein biomarkers by raman reporter indicator: Toward ultrasensitivity and universality. Biosens. Bioelectron..

[28] Arabi M., Ostovan A., Wang Y., Mei R., Fu L., Li J., Wang X., Chen L. (2022). Chiral molecular imprinting-based SERS detection strategy for absolute enantiomeric discrimination. Nat. Commun..

[29] Xing R., Wen Y., Dong Y., Wang Y., Zhang Q., Liu Z. (2019). Dual molecularly imprinted polymer-based plasmonic immunosandwich assay for the specific and sensitive detection of protein biomarkers. Anal. Chem..

