# Supplementary material for: Synthesis and Integration of Hybrid Metal Nanoparticles Covered with a Molecularly Imprinted Polymer Nanolayer by Photopolymerization
Source: Sensors (Basel). 2023 Apr 14;23(8):3995. doi: 10.3390/s23083995 (PMC10142421; doi:10.3390/s23083995)
Supplement: Supplementary file 1 [file sensors-23-03995-s001.zip › sensors-2320493-supplementary.pdf]

Supporting Information

Synthesis and integration of hybrid metal nanoparticles covered with a molecularly imprinted polymer nanolayer by photopolymerization

Amine Khitous[1,2], Celine Molinaro[1,2], Constance Thomas[1,2], Karsten Haupt[3], Olivier Soppera[1,2,*]

[1] *Université de Haute-Alsace, CNRS, IS2M UMR 7361, F-68100 Mulhouse, France*

[2] *Université de Strasbourg, France*

[3] *Université de Technologie de Compiègne, France*

* Contact: olivier.soppera@uha.fr

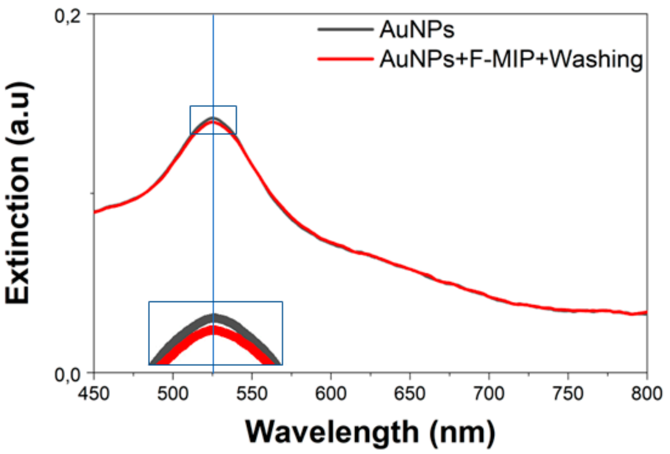

Figure S1: UV-vis spectra of bare AuNPs (**black**) and rinsed AuNPs after contact with the MIP-R6G formulation without irradiation (**red**)

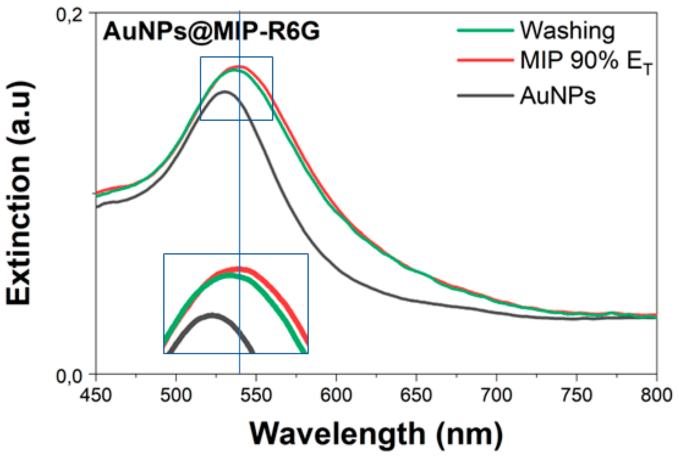

Figure S2: UV-vis spectra of bare AuNPs (**black**), functionalized by a layer of 90% E_T MIP-R6G (**green**) and rinsed after functionalization (**red**)

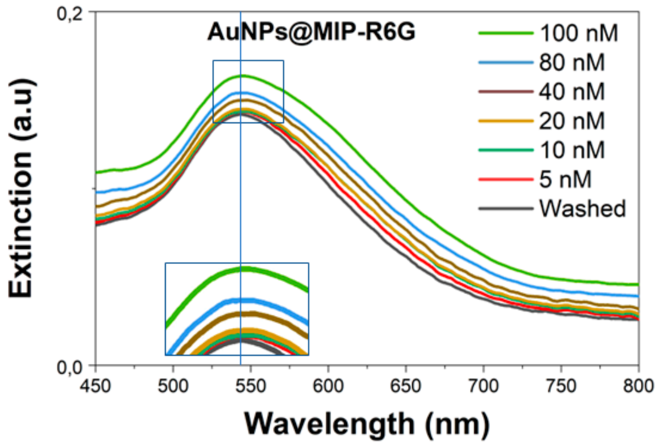

Figure S3: UV-vis spectra of AuNPs@MIP-R6G after incubation in R6G at different concentrations

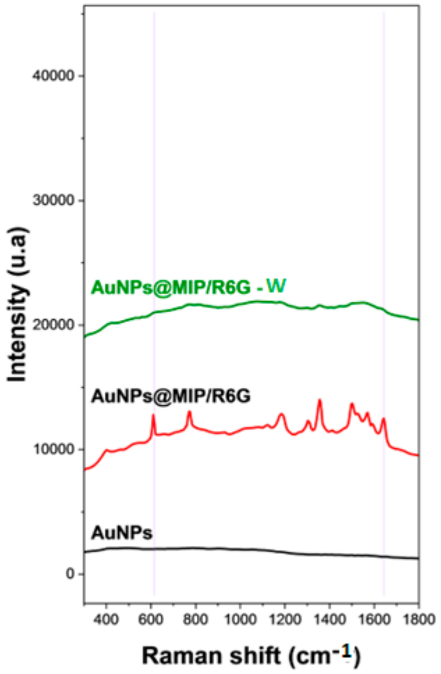

Figure S4: SERS spectra of **AuNPs**, AuNPs@MIP-R6G after **synthesis** and **washing**

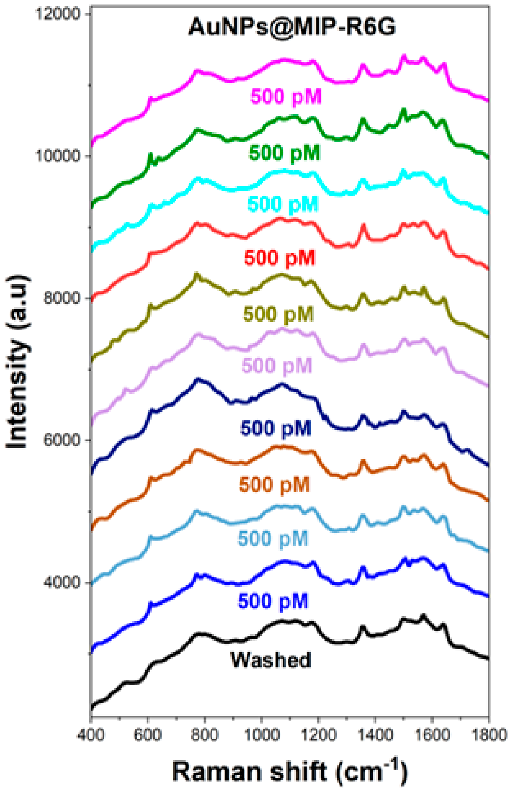

Figure S5: SERS spectra of AuNPs@MIP-R6G after 10 incubations in R6G at 500 pM (the sample is rinced between each incubation to remove the target)

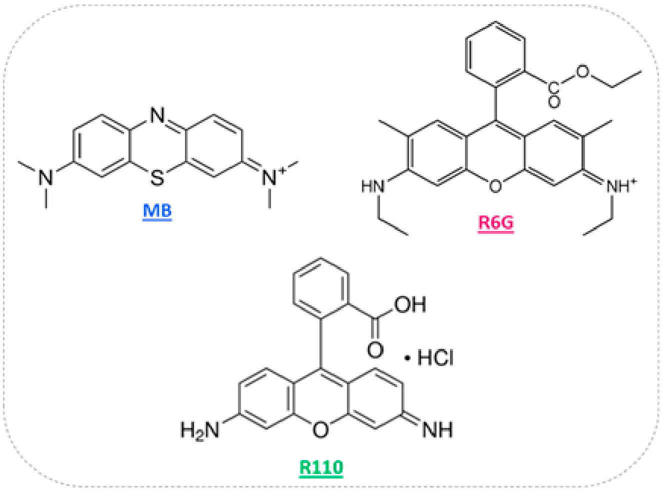

Figure S6: Molecular structures of: methylene blue (**MB**), rhodamine 6G (**R6G**) and rhodamine 110 (**R110**)
